# A chromosome-level genome assembly of rugged rose (*Rosa rugosa*) provides insights into its evolution, ecology, and floral characteristics

**DOI:** 10.1038/s41438-021-00594-z

**Published:** 2021-06-18

**Authors:** Fei Chen, Liyao Su, Shuaiya Hu, Jia-Yu Xue, Hui Liu, Guanhua Liu, Yifan Jiang, Jianke Du, Yushan Qiao, Yannan Fan, Huan Liu, Qi Yang, Wenjie Lu, Zhu-Qing Shao, Jian Zhang, Liangsheng Zhang, Feng Chen, Zong-Ming (Max) Cheng

**Affiliations:** 1 College of Horticulture, Nanjing Agricultural University, Nanjing, 210095 China; 2 Academy for Advanced Interdisciplinary Studies, Nanjing Agricultural University, Nanjing, 210095 China; 3 BGI-Shenzhen, Beishan Industrial Zone, Yantian District, Shenzhen, 518083 China; 4 Department of Biology, University of Copenhagen, Copenhagen, Denmark; 5 Grandomics Biosciences Co., Ltd, Wuhan, China; 6 School of Life Sciences, Nanjing University, Nanjing, China; 7 College of life science, Nantong University, Nantong, China; 8 Genomics and Genetic Engineering Laboratory of Ornamental Plants, College of Agriculture and Biotechnology, Zhejiang University, Hangzhou, China; 9 Department of plant sciences, University of Tennessee, Knoxville, TN USA

**Keywords:** Plant evolution, Chromosomes, DNA sequencing

## Abstract

*Rosa rugosa*, commonly known as rugged rose, is a perennial ornamental shrub. It produces beautiful flowers with a mild fragrance and colorful seed pods. Unlike many other cultivated roses, *R. rugosa* adapts to a wide range of habitat types and harsh environmental conditions such as salinity, alkaline, shade, drought, high humidity, and frigid temperatures. Here, we produced and analyzed a high-quality genome sequence for *R. rugosa* to understand its ecology, floral characteristics and evolution. PacBio HiFi reads were initially used to construct the draft genome of *R. rugosa*, and then Hi-C sequencing was applied to assemble the contigs into 7 chromosomes. We obtained a 382.6 Mb genome encoding 39,704 protein-coding genes. The genome of *R. rugosa* appears to be conserved with no additional whole-genome duplication after the gamma whole-genome triplication (WGT), which occurred ~100 million years ago in the ancestor of core eudicots. Based on a comparative analysis of the high-quality genome assembly of *R. rugosa* and other high-quality Rosaceae genomes, we found a unique large inverted segment in the Chinese rose *R. chinensis* and a retroposition in strawberry caused by post-WGT events. We also found that floral development- and stress response signaling-related gene modules were retained after the WGT. Two *MADS-box* genes involved in floral development and the stress-related transcription factors *DREB2A-INTERACTING PROTEIN 2* (*DRIP2*) and *PEPTIDE TRANSPORTER 3* (*PTR3*) were found to be positively selected in evolution, which may have contributed to the unique ability of this plant to adapt to harsh environments. In summary, the high-quality genome sequence of *R. rugosa* provides a map for genetic studies and molecular breeding of this plant and enables comparative genomic studies of *Rosa* in the near future.

## Introductions

*Rosa rugosa* is a perennial shrub tree that grows to 1–1.5 m tall and is native to Eastern Asia. It blooms and produces edible hips (the seed pods) in summer and early autumn. *R. rugosa* has been utilized in many ways. Because of its attractive pink flowers, *R. rugosa* is often used to create windbreaks and hedges. It has also been cultivated in North America and Europe as an introduced ornamental plant. The fruits of *R. rugosa* possess antioxidant activity and antibacterial activity due to their high contents of phenolic and flavonoid compounds and ascorbic acid^1,2^. It is able to control soil erosion and is planted along highways in Germany and Denmark^3^. Because of the high level of biosynthesis of pleasant volatile compounds in its flowers, *R. rugosa* has been used as an important source for the production of essential oil^4^. In breeding, *R. rugosa* has been widely used for breeding salt-resistant *Rosa* varieties. Although *R. rugosa* has many advantages, research on its molecular breeding and domestication has not even begun, partly due to the lack of high-quality genome sequences.

Also known as rugged rose, *R. rugosa* can adapt to many environmental conditions, such as salinity and alkaline soils, shade, frigid temperatures, drought, and high humidity. These excellent abilities make *R. rugosa* ideal for gene mining and molecular breeding to produce novel *Rosa* varieties. In some places, *R. rugosa* has become invasive^5^, attesting to its ability to adapt to new environments. However, the molecular mechanisms underlying this adaptability are largely unknown.

Following the rapid development of genome sequencing technologies and bioinformatic technologies, hundreds of angiosperm genomes have been reported^6–8^. The *Rosa* genus includes ~200 species with quite different morphological traits^9^. Within the *Rosa* genus, the first draft genome sequence of wild *Rosa multiflora* was released in 2018^10^. Since then, two chromosome-level genomes of *Rosa chinensis*, also known as Chinese rose, have been released^11,12^. For *R. rugosa*, only the chloroplast genome^13^ and mitochondrial genome^14^ have been reported. A high-quality genome sequence for *R. rugosa* would not only enable comparative genomic studies of *Rosa* species but also reveal the mechanisms underlying its ornamental traits, such as floral biology and its unique ecology.

Here, we report the first chromosome-level genome assembly of *R. rugosa*, relying on HiFi sequencing and Hi-C scaffolding technology. Based on this high-quality genome assembly, we studied the genomic structural differences between *R. rugosa* and *R. chinensis*. We also revealed the genetics responsible for floral biology. The mechanisms that account for its evolution and adaptation to harsh environments were explored here as well.

## Results and discussion

### Genome sequencing and assembly

**Fig. 1:**
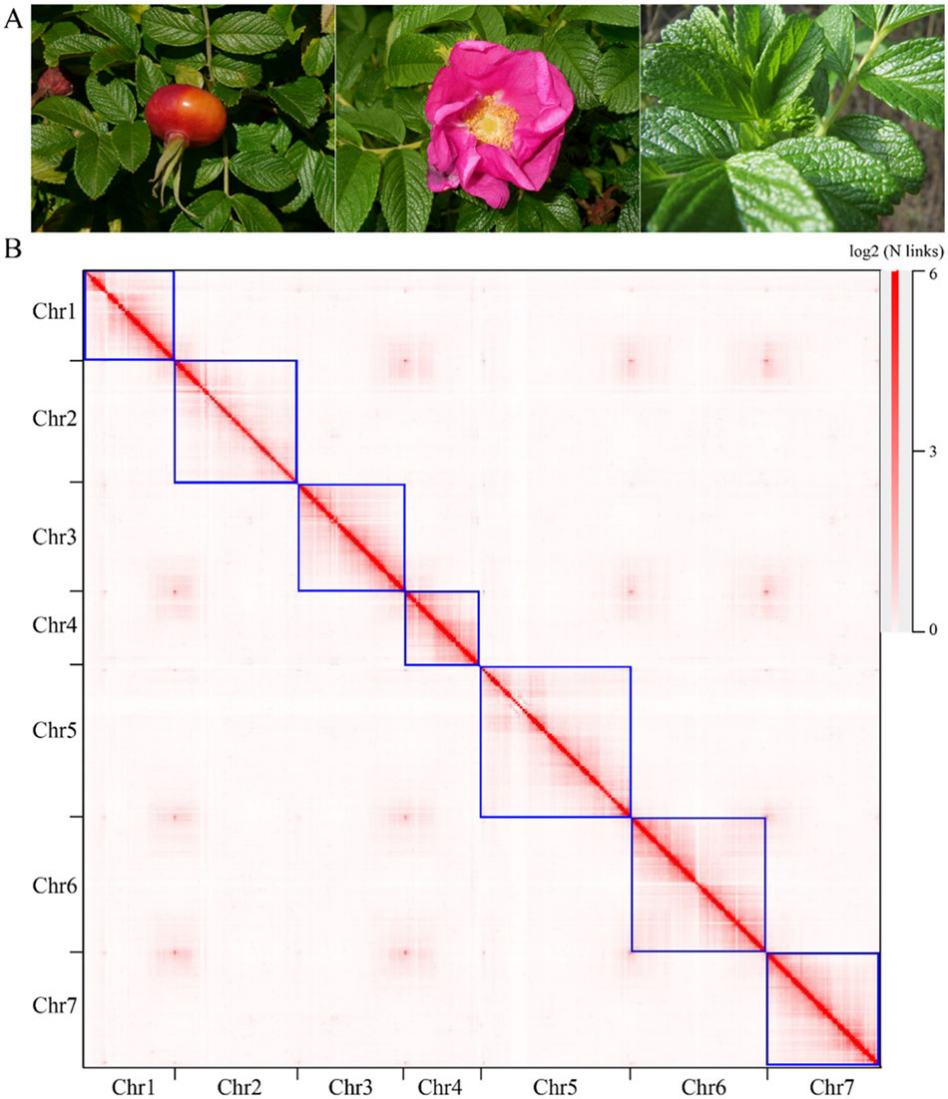
The Hi-C interaction heatmap of *R. rugosa* showing that the contigs were assembled into 7 chromosomes. **A** The fruit, flower, and leaf of *R. rugosa*. The tender leaf was sampled for genome sequencing. **B** The heatmap showed the 7 assembled chromosomes

We used a combination of sequencing technologies, including PacBio-CCS (HiFi), 10X genomics, and Hi-C, to construct the reference genome for *R. rugosa*. We obtained a total of 59.24 Gb HiFi clean data and 80.91 Gb 10X genomics clean data, respectively. We employed *K*-mer-based statistics to predict genome size, and it was estimated to be 454.78 Mb. The assembled genome is 382.64 Mb with a contig N50 of 15.36 Mb (Table 1), significantly longer than that in *R. chinensis* (contig N50 = 3.4 Mb)^12^ or woodland strawberry *Fragaria vesca* (contig N50 = 7.9 Mb)^15^. The GC content of the *R. rugosa* genome was 39.30% (Table 1), which was very similar to that of *F. vesca* (38.98%) and *R. chinensis* (38.84%). To assemble the contigs into chromosomes, we applied Hi-C sequencing technology and anchored 98.21% of the sequences onto 7 chromosomes (Fig. 1, Supplementary Table 1). Based on this high-quality genome assembly, we evaluated the genome completeness of *R. rugosa* using BUCSO with the embryophyte_odb10 database. The genome assembly completeness reached 93.2%, and the gene prediction completeness reached 94.4%. We further compared the completeness of *R. rugosa* with the released Rosaceae genomes of *R. chinensis*, strawberry (*F. vesca*), peach (*P. persica*), apple (*M. domestica*) and pear (*P. bretschneideri*). Their proportions were similar to those of *R. rugosa* (Supplementary Table 2), indicating the high quality of our genome assembly.

**Table 1:** Statistics of the *R. rugosa* genome assembly and annotation

Feature	Value
Raw data of PacBio-HiFi sequencing (Gb)	59.24
Raw data of 10X Genomics (Gb)	80.91
Raw data of Hi-C sequencing (Gb)	150.6
Estimated genome size (Mb)	454.78
Assembled contigs (Mb)	382.64
Contig N50 (Mb)	15.36
Number of contig	105
Largest contig (Mb)	31.80
Total size of chromosome (Mb)	375.79
GC content (%)	39.30
Heterozygosity (%)	0.71
Number of genes	39,704

### Genome components

**Fig. 2:**
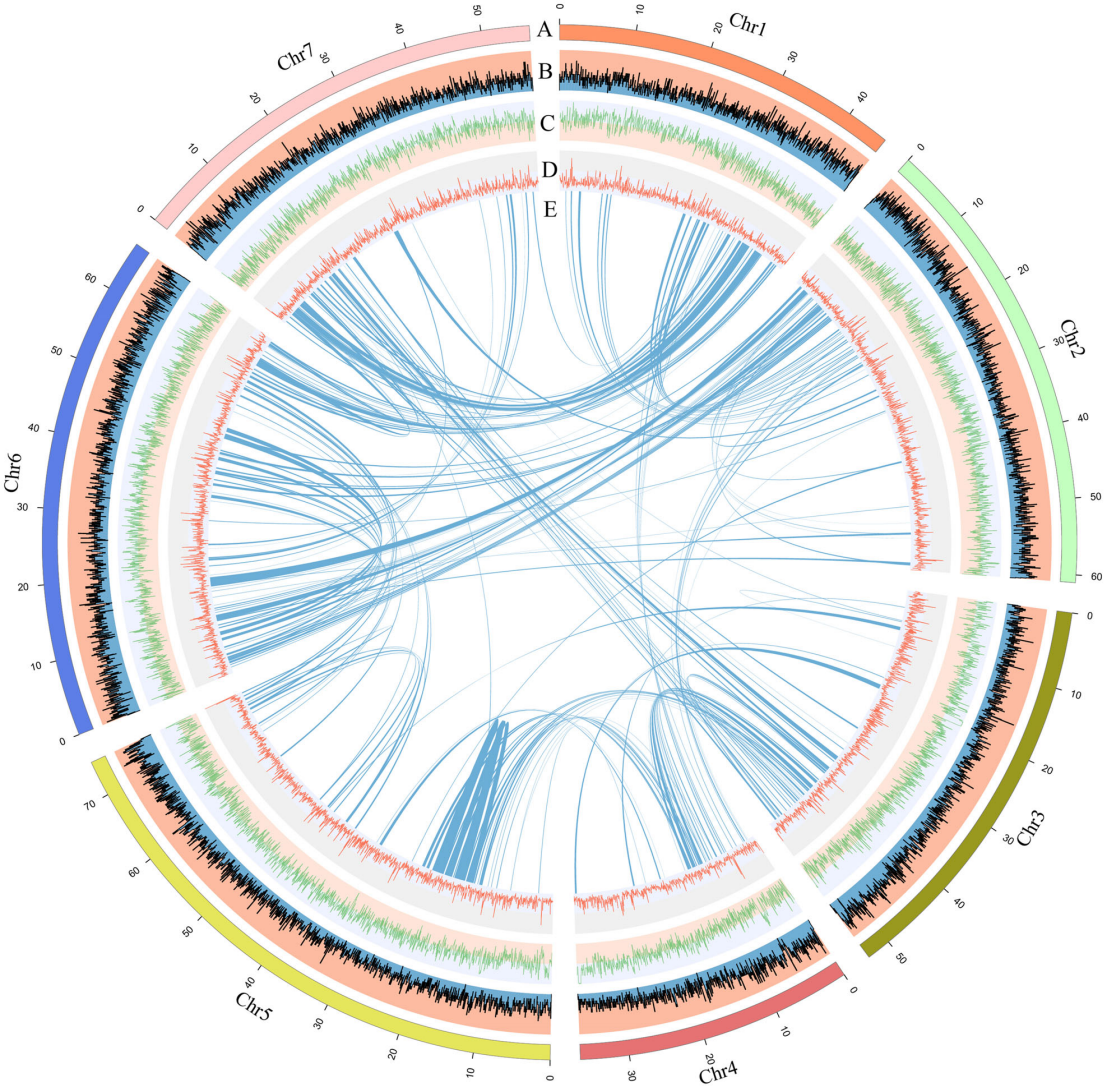
The high-quality genome assembly of *R. rugosa* allows the visualization of genomic details. **A** The 7 chromosomes. **B** Gene density (window size = 50 kb), **C** LTR density (window size = 50 kb). **D** GC content distribution (windows size = 50 kb). **E** Synteny blocks in *R. rugosa*

The *R. rugosa* genome was composed of 50.27% repetitive sequences (Table 2). Most of these repetitive sequences are long terminal repeats (LTRs), including *G*ypsy and *Copia*, accounting for 26.75% of the total genome. The proportion of LTRs in *R. rugosa* was much greater than that in *Fragaria* spp. such as *F. vesca* (~16%)^16^ and *F. nilgerrensis* (16.5%)^17^ but slightly less than that in *R. chinensis* (28.3%)^12^, suggesting the rapid evolution of LTRs in Rosaceae plants. The *R. rugosa* genome encodes 39,704 protein-coding genes, close to the number in *R. chinensis*^12^. Moreover, we annotated 37.32%, 87.58%, and 23.03% of genes using the Gene Ontology (GO), Kyoto Encyclopedia of Genes and Genomes (KEGG) and Clusters of Orthologous Groups (COG) databases (Supplementary Figs. 1, 2, 3). We mapped the genes and repetitive elements to the 7 chromosomes (Fig. 2).

**Table 2:** Repeat sequences in the *R. rugosa* genome

	Type	Number of elements	Length occupied (bp)	Percentage of sequence (%)
Retroelements		111,329	118367,513	30.04
	SINEs:	5594	793,802	0.2
	Penelope	30	19,393	0
	LINEs:	20,751	12,160,801	3.09
	L2/CR1/Rex	457	449,390	0.11
	L1/CIN4	20,036	11,601,916	2.94
	LTR elements:	84,984	105,412,910	26.75
	BEL/Pao	53	14,816	0
	Ty1/Copia	32,581	38,364,733	9.74
	Gypsy/DIRS1	50,138	65,699,666	16.67
	Retroviral	255	74,660	0.02
DNA transposons		83,506	25,795,514	6.55
	hobo-Activator	21,137	6,286,361	1.6
	Tc1-IS630-Pogo	204	46,086	0.01
	PiggyBac	376	130,940	0.03
Rolling-circles		4111	2,498,640	0.63
Unclassified:		172,477	46,290,831	11.75
Total repeats:			190,453,858	48.33
Small RNA:		5982	926,674	0.24
Satellites:		875	279,368	0.07
Simple repeats:		103,313	3,857,918	0.98
Low complexity:		16,875	814,967	0.21

### Evolution of the *R. rugosa* genome

**Fig. 3:**
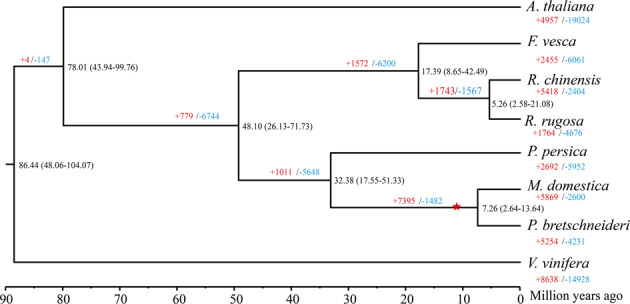
A phylogenomic species tree of *R. rugosa* and eight other representative species. This tree was constructed using 321 stringent single-copy nuclear protein-coding genes, showing gene family contraction and expansion. The numbers above the branches represent the number of gene families with either expansion (red) or contraction (blue). The numbers at the node indicate divergence time and 95% confidence interval. The species used in the tree are *Arabidopsis thaliana*, *Fragaria vesca*, *Rosa chinensis*, *Rosa rugosa*, *Prunus persica*, *Malus domestica*, and *Pyrus bretschneideri*. The red star indicates the whole-genome duplication in the ancestor of apple (*M. domestica*) and pear (*P. bretschneideri*)

To study the evolution of the *R. rugosa* genome, we constructed a species tree of *R. rugosa* and representative Rosaceae species using phylogenomics. We obtained 321 high-confidence single-copy nuclear genes across 8 eudicot species. *R. rugosa* is closely related to *R. chinensis*, diverging ~5.26 million years ago (Fig. 3). Although they are close relatives in the *Rosa* genus, the gene orthogroups differ greatly in these two species, gaining 5418 and 1764 in *R. chinensis* and *R. rugosa*, respectively, and losing 2404 and 4676 in *R. chinensis* and *R. rugosa*, respectively. The genus *Rosa* could be divided into two groups: group I: Pimpinellifoliae+Rosa+Carolinae and group II: Gallicanae+Synstylae+Chinenses+Laevigatae+Caninae+Banksianae+Microphyllae+Bracteatae. This significant orthogroup difference may be because *R. rugosa* belongs to Group I and *R. chinensis* belongs to Group II^18^.

We then explored the orthogroup variations between *R. rugosa* and *R. chinensis*. We studied both the contracted and expanded orthogroups in these two species (Table 3). We showed that *R. rugosa* lost several orthogroups, including OG0000650 (aldolase superfamily), OG0000325 (aminotransferase-like), OG0001051 (IBR domain-containing), OG0000709 (NB-ARC domain-containing disease resistance), and OG0000761 (NB-ARC domain-containing disease resistance), but had more NB-ARC domain-containing disease resistance protein genes than OG0000869.

**Table 3:** The expansion and contraction of orthogroups between *R. rugosa* and *R. chinensis*

Family	*R. rugosa*	*R. chinensis*	Expansion or contraction	Annotation
OG0000000	338	7	331	Ribonuclease H-like superfamily
OG0000172	60	0	60	Retroviridae gag-proteins
OG0000013	80	24	56	Ribonuclease H-like superfamily
OG0000189	55	1	54	Retroviridae gag-proteins
OG0000016	74	20	54	Ribonuclease H-like superfamily
OG0000055	69	18	51	Ribonuclease H-like superfamily
OG0000044	63	14	49	DNA/RNA polymerases superfamily protein
OG0000180	48	9	39	DNAse I-like superfamily protein
OG0000250	42	4	38	Cysteine-rich receptor-like protein kinase
OG0000516	39	1	38	Zinc knuckle (CCHC-type) family protein
OG0000177	47	10	37	Ribonuclease H-like superfamily
OG0000564	37	0	37	Ribonuclease H-like superfamily
OG0000174	43	7	36	Cysteine-rich receptor-like protein Kinase
OG0000517	38	2	36	Ribonuclease H-like superfamily
OG0000126	40	5	35	Cysteine-rich receptor-like protein kinase
OG0000098	45	10	35	DNA/RNA polymerases Superfamily protein
OG0000652	36	1	35	Ribonuclease H-like superfamily
OG0000384	35	1	34	Cysteine-rich RECEPTOR-like kinase
OG0000028	59	25	34	MuDR family transposase
OG0000052	44	10	34	Ribonuclease H-like superfamily
OG0000116	46	14	32	WUS/WUSCHEL
OG0000869	32	1	31	NB-ARC domain-containing Disease resistance protein
OG0000100	36	5	31	Ribonuclease H-like superfamily
OG0000514	35	4	31	Ribonuclease H-like superfamily
OG0000288	32	3	29	zinc knuckle (CCHC-type) family protein
OG0001051	0	31	−31	IBR domain-containing protein
OG0000450	3	35	−32	NB-ARC domain-containing Disease resistance protein
OG0000020	22	54	−32	TIR-NBS-LRR class
OG0000762	1	34	−33	NB-ARC domain-containing Disease resistance protein
OG0000761	0	35	−35	NB-ARC domain-containing Disease resistance protein
OG0000049	31	66	−35	TIR-NBS-LRR class
OG0000709	0	36	−36	NB-ARC domain-containing Disease resistance protein
OG0000650	0	37	−37	Aldolase superfamily protein
OG0000041	21	63	−42	Nuclease
OG0000147	1	47	−46	ANTHRANILATE SYNTHASE BETA SUBUNIT 1
OG0000325	0	48	−48	Aminotransferase-like, plant mobile domain family protein
OG0000009	13	66	−53	Leucine-rich repeat (LRR) family

**Fig. 4:**
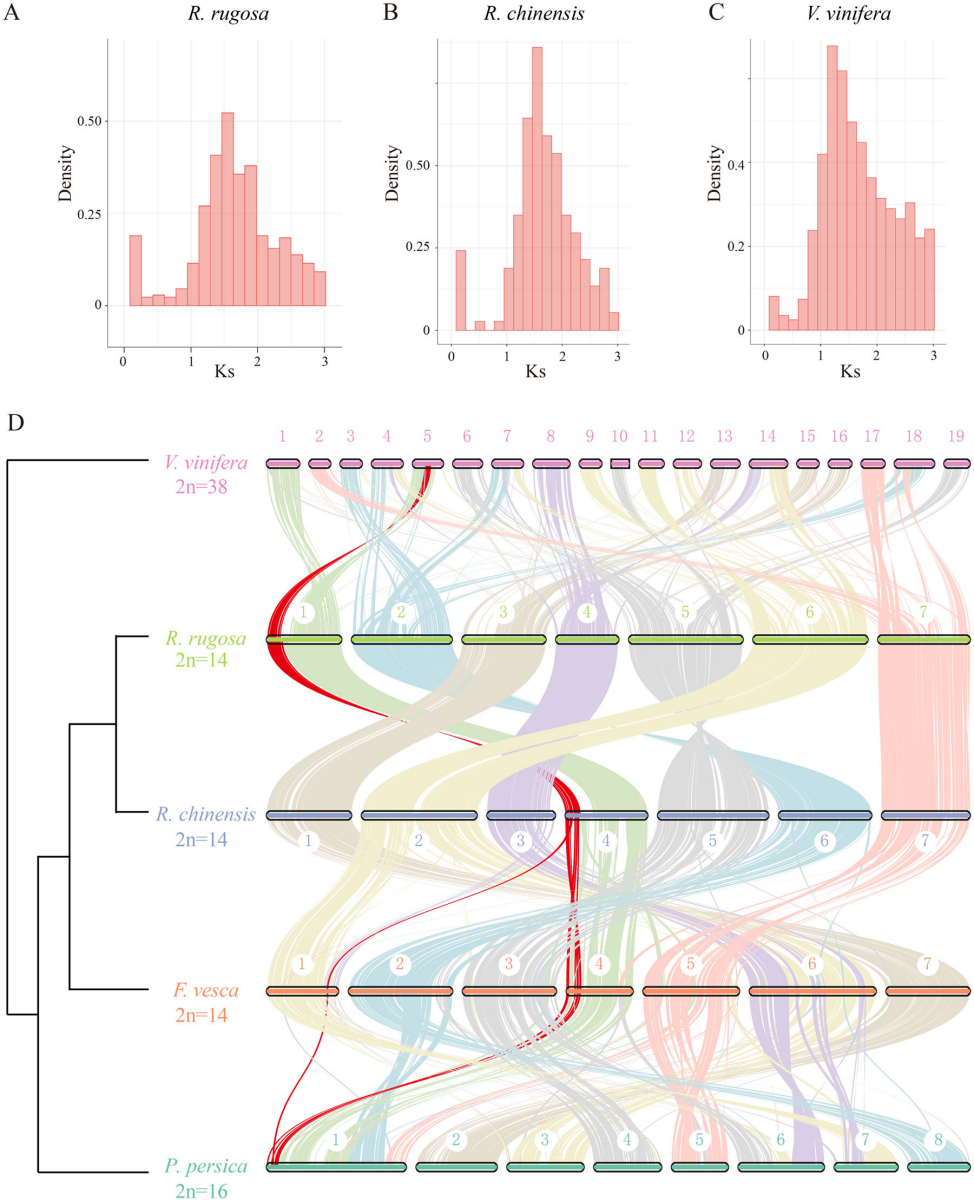
*Rosa rugosa* experienced only the core eudicot-specific gamma whole-genome triplication, with no recent polyploidization. **A** *K*s distribution of *R. rugosa* paralogs. **B** *K*s distribution of *R. chinensis* paralogs. **C** *K*s distribution of *P. persica* paralogs. **D** Cross-species comparison using several eudicots, including grape (*V. vinifera*), *R. rugosa*, *R. chinensis*, strawberry (*F. vesca*), and peach (*P. persica*). The red lines indicate genomic shuffling across these Rosaceae species using grapes as an outgroup

The publications of hundreds of angiosperm genomes^6^ has revealed that polyploidization events have occurred frequently, with at least four waves^19^, contributing to the genomic materials for innovation^20^. We calculated the gene *K*s values in *R. rugosa*, *R. chinensis*, and *Vitis vinifera*. We found that their shared feature is a single peak at 1.3-1.5 (Fig. 4A–C). We then compared the whole-genome syntenic patterns and still did not find any recent WGD. These results show that the *Rosa* species experienced only the eudicot-specific WGT event, similar to grapes^21^. This result is consistent with previous reports for other *Rosa* species^22^.

We compared the syntenic patterns of *R. rugosa* with those of other representative species (Fig. 4D). We showed that *R. rugosa* has very conserved syntenic relationships with grape. For example, VvChr1 and VvCh5 correspond to RrChr1, VvChr9, and VvChr11, and half of VvChr14 matches RrChr6 (Fig. 4D, Supplementary Fig. 4). In the genomes of *R. rugosa* and *R. chinensis*, every chromosome matched each other well. However, when compared with two other Rosaceae species, namely, peach (*P. persica*) and wild strawberries (*F. vesca*), we found that a large segment composed of 10.44 Mb of chromosome was reversed in *R. chinensis* but in the exact same order in other species (Fig. 4D, Supplementary Fig. 5). In addition, we found that a segment 1.56 Mb in length was translocated in *F. vesca*. These results suggest that genomes within the *Rosa* genus are very conserved in terms of synteny and that small genetic changes could contribute to morphological variations.

Since we did not find significant expansion or loss of genes related to the salt stress response or water stress in *R. rugosa* compared to *R. chinensis* (Table 3), we then investigated the contribution of WGT to *R. rugosa*.

**Fig. 5:**
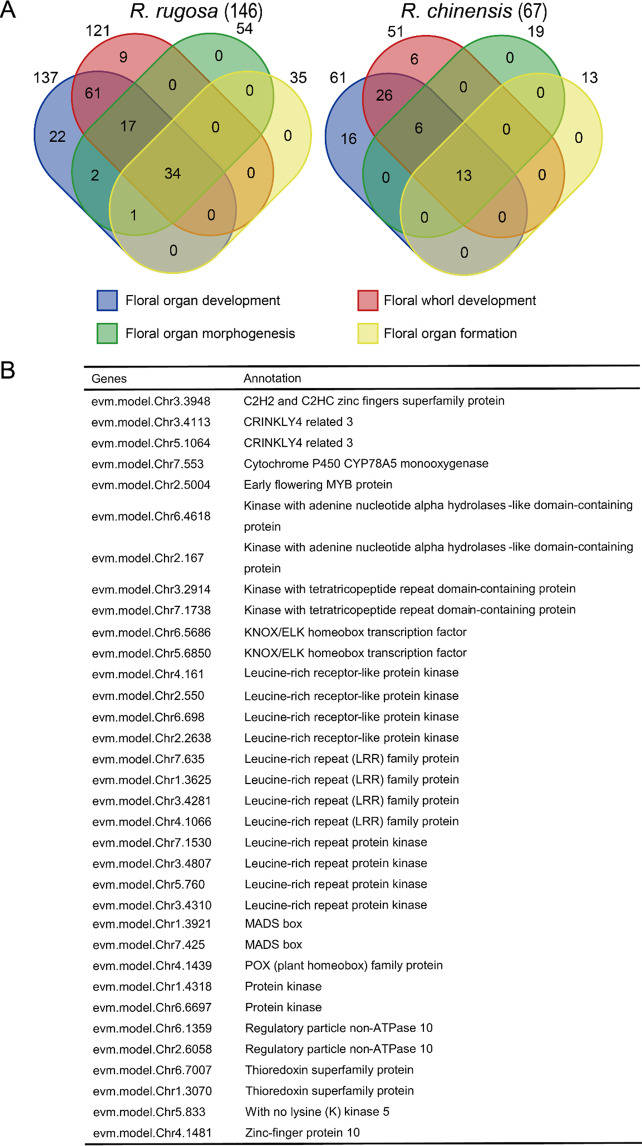
The floral developmental genes were retained after gamma WGT in *R. rugosa*. **A** The Venn diagram shows the distribution of genes involved in floral organ development, floral whorl development, floral organ morphogenesis, and floral organ formation from *R. chinensis* and *R. rugosa*. **B** Annotation of the 34 genes involved in four aspects of floral development in *R. rugosa* identified a series of kinase and transcription factor genes

We studied the WGT and its contribution to floral evolution in *R. rugosa* and *R. chinensis*. *R. rugosa* has large, pink, and fragrant flowers. We analyzed the genes retained after WGT to determine whether floral genes could have expanded after this ancient polyploidization event. Gene Ontology annotation of all *R. rugosa* protein-coding genes showed that 146 genes, compared to 67 genes in *R. chinensis*, were involved in floral organ development (Fig. 5A, Supplementary Figs. 6, 7), suggesting that *R. rugosa* retained many more genes for floral-related traits. Floral organ development was divided into four categories, including floral organ development, floral whorl development, floral organ morphogenesis and floral organ formation, according to the agriGO analyses. Among them, 34 genes, including kinase proteins (LRR kinases) and transcription factors (*KNOX/ELK*, *MYB*, *zinc finger* and *MADS-box*), were involved in all four aspects in *R. rugosa* (Fig. 5B). However, only 13 genes were involved in all four aspects in *R. chinensis* (Supplementary Table 3). Then, we compared the floral organ determination genes and the *MADS-box* genes in *R. rugosa*, *R. chinensis*, and *A. thaliana*. We found a total of 92 MADS-box genes in *R. rugosa*, slightly more than that in *R. chinensis* (84 MADS-box genes) (Supplementary Fig. 8). The *S*-locus of *R. rugosa* was investigated for the first time and compared with other Rosaceae species (Supplementary Fig. 9). The results showed that there were 19 *F-box* genes and one *S-RNase* gene in *R. rugosa*. Unlike *Prunus* spp., *R. rugosa*’s S-locus size was similar to that in *Maleae* spp., suggesting that the self-incompatibility recognition mechanism was closer to or belonged to the multifactor recognition model.

*R. rugosa* plants are economically important partly due to the high essential oil production of their flowers. Monoterpenes are the main constituents of essential oils, accounting for 50–70% of the total content^23,24^. Due to the lack of genome sequences, only a fraction of genes could be identified using transcriptomes or comparative genomic studies^24^. Here, a total of 53 terpene synthases (TPSs), which are key genes responsible for terpene biosynthesis, were identified from the genome of *R. rugosa* (Supplementary Fig. 10). The RrTPSs were distributed into five subfamilies (TPS-a, b, c, g and e/f) based on clustering with TPS identified from model angiosperm species^25^. Eighteen and four RrTPS genes were found to belong to the TPS-g and TPS-b subfamilies, respectively. Because TPS-g and TPS-b are mainly involved in monoterpene biosynthesis, these 22 RrTPS genes are the main candidates responsible for the high-level production of monoterpenes in essential oil. Twenty-six RrTPS genes were identified to be members of the TPS-a subfamily with putative sesquiterpene synthase functions. In addition, the TPS family in *R. rugosa* contains two members in the TPS-c subfamily and 3 members in the TPS-e/f subfamily. Further phylogenetic analysis indicated that each RrTPS gene, a member of TPS-g, has corresponding orthologs in the genome of *R. chinensis* (Supplementary Fig. 10), suggesting a close evolutionary relationship between the two TPS families from *R. rugosa* and *R. chinensis*.

**Fig. 6:**
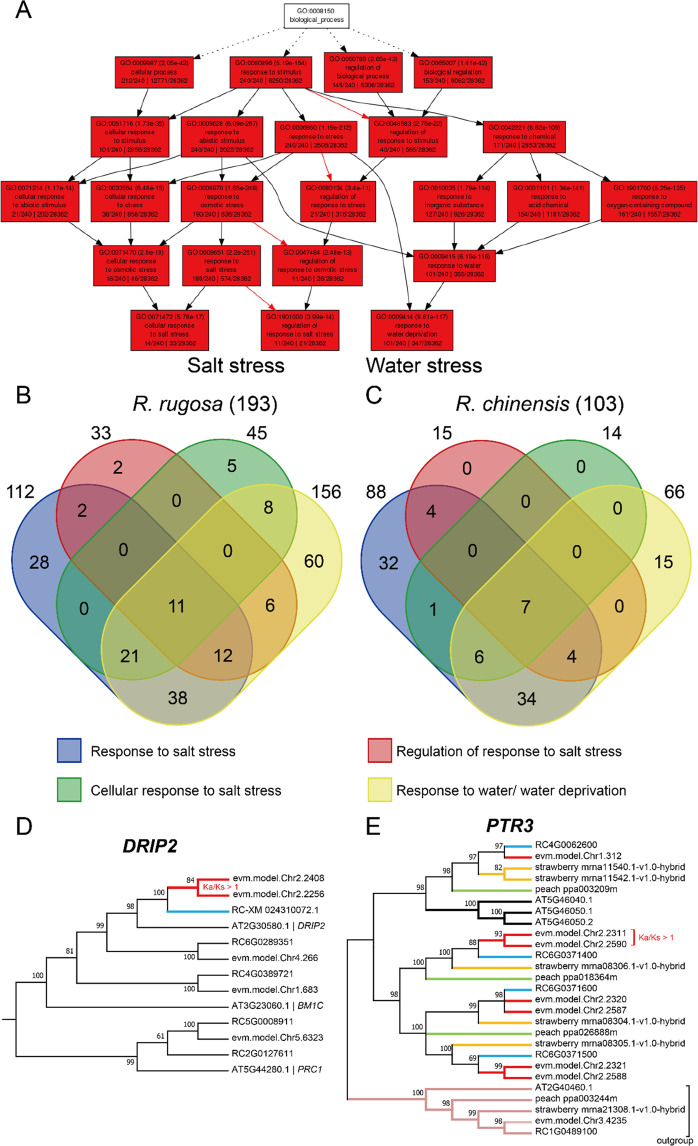
Abiotic stress-related genes were enriched in *Rosa rugosa*. **A** The agriGO modules of salt stress- and water stress-related genes predicted using *Arabidopsis* orthologs of *R. rugosa* duplicated genes after WGT. **B**, **C** Venn clustering of 193 and 103 abiotic stress-related genes from *R. rugosa* and *R. chinensis*, respectively. **D, E** The DRIP2 genes have two paralogs in *R. rugosa* but one in *R. chinensis* and *Arabidopsis*. The PTR3 genes have 7 paralogs in *R. rugosa* but 4 in *R. chinensis*. The DRIP2 paralogs and two PTR3 paralogs in *R. rugosa* have been subjected to strong positive selection pressure

*R. rugosa* can adapt to drought, salinity, and alkaline soils and can even become invasive in some places^3^. However, *R. chinensis* does not have such abilities. By pathway enrichment of all *R. rugosa* genes (Supplementary Fig. 2), we showed that 850 genes in *R. rugosa* were involved in environmental adaptation. To trace the origin and evolution of these stress-related genes, we found that two pathways, salt stress and water stress (water deprivation or drought), were significantly retained and enriched after WGT (Fig. 6A**for** ***R. rugosa***, Supplementary Fig. 11**for** ***R. chinensis***). In each module of *R. rugosa*, the number of genes was significantly higher than that in *R. chinensis*. Furthermore, we constructed a Venn diagram (Fig. 6B, C) to show the genes that might be involved in cross talk related to these abiotic stresses. Eventually, we found 11 and 7 genes in *R. rugosa* and *R. chinensis* that were predicted to be involved in these four abiotic stress responses, respectively (Supplementary Table 4, Supplementary Table 5). Notably, among these module genes, we found that two paralogs of *DREB2A-INTERACTING PROTEIN 2* (*DRIP2*) in *R. rugosa* had been subjected to positive selection pressure (Fig. 6D, Supplementary Table 6). Furthermore, we found two drought/water stress-related *DRIP2* genes in *R. rugosa* but only one in *R. chinensis* or *Arabidopsis*, with potential gene neofunctions in *R. rugosa*’s adaptation to stressful environments. Meanwhile, we found that the number of *PTR3* genes, which encode dipeptide and tripeptide transporters involved in responses to high NaCl concentrations, expanded to 7 in *R. rugosa* but only four in *R. chinensis*, 5 in *F. vesca*, 3 in *P. persica* and 3 in *A. thaliana*. Two *PTR3* (*PEPTIDE TRANSPORTER 3*) genes under positive selection pressure were detected (Fig. 6E, Supplementary Table 6). Therefore, these genes might provide *R. rugosa* with its unique ability to adapt to high salinity environments and water stresses.

**Fig. 7:**
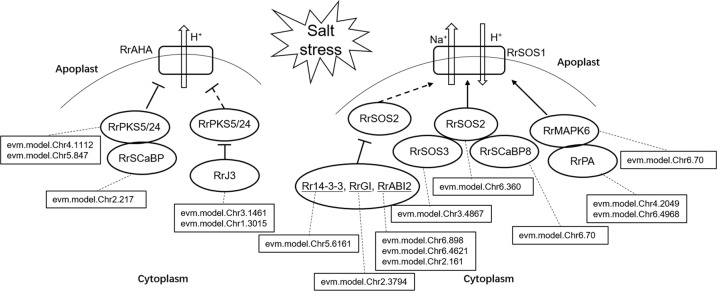
The deduced salt stress response signaling pathway in *R. rugosa*. The related *R. rugosa* genes were listed in rectangular boxes

Finally, as shown in Fig. 7, we constructed the salt stress response pathway of *R. rugosa*. Meanwhile, we compared the differences in the number of genes between *A. thaliana*, *R. rugosa* and *R. chinensis* (Supplementary Table 7). There was no difference in the number of genes among these sampled species, suggesting that *R. rugosa* did not cope with salt stress using gene dosage, but rather using transcription-, translation-, or metabolome-level regulation.

## Conclusions

As a popular ornamental plant, *R. rugosa* is widely cultivated. The flowers of *R. rugosa* have been utilized for essential oil production and dried to produce flower tea. The economic value of this plant will certainly grow if molecular breeding accelerates the production of novel cultivars with optimized essential oil content and improved floral traits. A high-quality reference genome will provide a map for the identification of genes responsible for key agronomic traits and provide insights into how rugosa rose evolved during its long evolutionary history. This study provides for the first time the valuable resource of a *R. rugosa* genome for the rose research community. Through analysis of the genome sequence of *R. rugosa* and comparative genomic analyses, we provide novel insights into the biology, ecology and evolution of *R. rugosa* from three main perspectives. From the perspective of structural genomics, we show a large reversed segment in *R. chinensis* and a translocation in strawberry. From the perspective of floral biology, we found that more *MADS-box* genes were retained in *R. rugosa* than in *R. chinensis*, suggesting their potential roles in floral development in *R. rugosa*. From the perspective of stress biology, a number of stress-related genes were found to be specifically expanded and retained in *R. rugosa*, potentially contributing to its adaptation to stressful environments.

## Materials and methods

### Plant samples and DNA/RNA extraction

The *R. rugosa* plants were sampled from Nanjing Agricultural University. For genome sequencing, we collected mature healthy *R. rugosa* leaves. For transcriptome sequencing, the roots, stems, and leaves of *R. rugosa* were obtained. All samples were quickly frozen in liquid nitrogen and stored in a −80 °C freezer. We used a QIAGEN® Genomic DNA extraction kit (Cat#13323, QIAGEN) to extract genomic DNA according to the standard operating procedure provided by the manufacturer. We isolated total RNA for RNA sequencing by TRIzol reagent according to the manufacturer’s instructions.

### Sequencing and library construction

We used a total of 15 µg genomic DNA to construct a SMRTbell target size library for PacBio-HiFi sequencing according to a standard protocol. We sheared genomic DNA to the expected size of fragments for sequencing on a PacBio Sequel II instrument with Sequencing Primer V2 and Sequel II Binding Kit 2.0 in Grandomics. To construct the Hi-C library, we digested cross-linked chromatin into units with Dpn II, marked by incubation with biotin-14-dCTP and ligated the units by biotinylation. Finally, the ligated genomic DNA was sheared to 100 bp by StLFT technology and sequenced using the DIPSEQ platform at BGI, China. One microgram of sample RNA was used to build an RNA library with a TruSeq RNA Library Preparation Kit (Illumina, USA) following the manufacturer’s recommendations.

### Genome assembly and quality evaluation

Approximately 59.2 Gb of raw HiFi sequencing reads was obtained from the rosa DNA library. We first used HiCanu v2.2.1^26^ for preliminary assembly of the rosa genome. Then, Redundans v 0.14a^27^ was performed to remove the redundant sequences. A total of 150.6 Gb of Hi-C data were obtained to anchor the contig onto the chromosome. We aligned Hi-C reads to assembly by BWA v 0.7.17-r1188^28^. Next, the draft assembly genome was scaffolded with Hi-C reads by 3D-DNA v180114^29^. Then, Juicer was used to filter the sequence and cluster it, and the Juicerbox tool^30^ was applied to manually adjust chromosome construction. We finally anchored the scaffolds on seven chromosomes. In addition, the BUSCO v3.0.2^31^ pipeline was used to assess the completeness and accuracy of the *R. rugosa* genome with the embryophyte_odb10 dataset, which contains 1614 BUSCO gene sets.

### Genome annotation

To annotate the repeat sequence in *R. rugosa*, RepeatModeler v2.0.1^32^ and RepeatMasker v4.1.0^33^ were searched using Repbase TE libarary (v2018.10.26) from the Repbase server (https://www.girinst.org/repbase/)^34^. To predict the protein-coding gene *R. rugosa*, we combined de novo gene prediction, homology-based prediction and RNA-seq-based prediction. SNAP v2006.07.28^35^ and AUGUSTUS v3.3.3^36^ were used for de novo prediction with the parameter file trained on *F. vesca*, *M. domestica*, *P. persica*, *P. bretschneideri*, *R. chinensis* and *R. occidentalis*. For homology-based and RNA-seq-based gene identification, *F. vesca*, *M. domestica*, *P. persica*, *P. bretschneideri*, *R. chinensis* and *R. occidentalis* genomes were searched. Then, we mapped RNA-seq data to the genome by Hisat2 v2.2.1^37^ and obtained gene models with SAMtools v1.7.1^38^. These transcripts and the genes from the six homologous species were analyzed with GeMoMa v1.6.4 software to identify protein-coding genes^39^. Finally, we merged the gene models with EVidenceModeler V1.1.1^40^ from SNAP v2006.07.28, AUGUSTUS V3.3.3 and GeMoMa v1.6.4. We annotated the COG/KOG^41^, Gene Ontology^42^ and KEGG pathways^43^ of rosa protein sequences on the eggNOG-mapper online website (http://eggnog-mapper.embl.de/) and used HMMER v3.3.1^44^ with the Pfam database^45^ to identify the functions of all proteins.

### Construction of phylogenetic trees and estimation of divergence times

We used OrthoFinder v2.4.0^46^ to generate clusters of gene families from rugged rose (*R. rugosa*), Arabidopsis (*A. thaliana*), strawberry (*F. vesca*)*, M. domestica, P. persica, P. bretschneideri, R. chinensis* and *V. vinifera*. We aligned the single-copy proteins generated from OrthoFinder v2.4.0^46^ by MUSCLE v3.8.1551^47^. Based on the single-copy nuclear genes from the MUSCLE results, we used RAxML v8.2.12^48^ and ASTRAL-II v5.7.3^49^ to construct the phylogenetic tree with the maximum-likelihood method. Then, we used the MCMCTree pipeline of the PAML v4.9^50^ program to calculate the divergence times of the eight species. We marked the split times of *Rosids* and *Rosaceae* that were downloaded from the TimeTree website (http://timetree.org/).

### Gene family expansion and contraction

Based on the gene family and gene count statistics of OrthoFinder v2.4.0, we used CAFÉ v4.2.1^51^ to determine the expansion and contraction gene families of *R. rugosa*, *A. thaliana*, *F. vesca, M. domestica, P. persica, P. bretschneideri, R. chinensis* and *V. vinifera* with a *p* value < 0.01.

### Synteny and WGD

To find the synteny blocks between *R. rugosa*, *R. chinensis* and *V. vinifera*, the python version of MCScan (JCVI v1.1.7)^52^ was applied to compare proteins to proteins. We set 30 genes as the minimum in a syntenic region. Furthermore, we constructed a Circos map by Circos v0.52^53^. To analyze whole-genome duplications in rosa, we calculated and mapped the *K*s values and distribution by wgd v1.1.0^54^.

### Genes under positive selection

To analyze positively selected genes, we chose *F. vesca, M. domestica, P. persica, P. bretschneideri* and *R. chinensis* to identify orthologs by WGD. ParaAT v2.0^55^ and KaKs_Calculator v2.0^56^ were used to detect the genes under positive selection. Next, we used BLASTP to search for homologous genes between *R. rugosa* and *A. thaliana*. AgriGO v2.0^57^ was used to annotate the GO, and we drew a Venn diagram on an online website (http://bioinformatics.psb.ugent.be/webtools/Venn/).

## Supplementary information

A chromosome-level genome assembly of rugged rose (Rosa rugosa) provides insights into its evolution, ecology, and floral characteristics

## Data Availability

All the raw data, as well as genome sequences, protein sequences, CDSs, and GFF files, could be found in our eplant database (http://eplant.njau.edu.cn).
