# Supplementary material for: A chromosome-level genome assembly of rugged rose (Rosa rugosa) provides insights into its evolution, ecology, and floral characteristics
Source: Hortic Res. 2021 Jun 18;8:141. doi: 10.1038/s41438-021-00594-z (PMC8213826; doi:10.1038/s41438-021-00594-z)
Supplement: Supplementary file 1 — A chromosome-level genome assembly of rugged rose (Rosa rugosa) provides insights into its evolution, ecology, and floral characteristics [file 41438_2021_594_MOESM1_ESM.docx]

**[Supplementary files]**

**[Title] A chromosome-level genome assembly of rugged rose (*Rosa rugosa*) provides insights into its evolution, ecology, and floral characteristics**

**[Running title] The *Rosa rugosa* genome sequence**

Fei Chen1#*, Liyao Su1#, Shuaiya Hu1, Jiayu Xue1, Hui Liu1, Guanhua Liu1, Yifan Jiang1, Jianke Du1, Yushan Qiao1, Yannan Fan2,3, Huan Liu2,3, Qi Yang4, Wenjie Lu4, Zhuqin Shao5, Jian Zhang6, Liangsheng Zhang7, Feng Chen8, Zong-Ming (Max) Cheng1*

1, College of Horticulture, Nanjing Agricultural University, Nanjing 210095, China

2, BGI-Shenzhen, Beishan Industrial Zone, Yantian District, Shenzhen 518083, China

3, Department of Biology, University of Copenhagen, Copenhagen, Denmark

4, Grandomics Biosciences Co., Ltd, Wuhan, China

5, College of life science, Nanjing University, Nanjing, China

6, College of life science, Nantong University, Nantong, China

7, Genomics and Genetic Engineering Laboratory of Ornamental Plants, College of Agriculture and Biotechnology, Zhejiang University, Hangzhou, China

8, Department of plant sciences, University of Tennessee, Knoxville, TN, USA

*Correspondence: Zong-Ming (Max) Cheng (zcheng@utk.edu)

# Co-first author

*Co-corresponding author

**Supplementary Table 1. Summary of *R. rugosa’s* chromosome details.**

| Pseudochromosome | Sequence length (bp) |
| --- | --- |
| chr1 | 45701128 |
| chr2 | 61014187 |
| chr3 | 53196450 |
| chr4 | 36378635 |
| chr5 | 74700907 |
| chr6 | 66736445 |
| chr7 | 56320814 |
| Total Coverage of the genome | 98.21% |

**Supplementary Table 2. Genome BUSCO results of *R. rugosa* and other representative plant species**

**Supplementary Table 3. The common floral developmental genes in *Rosa chinensis* after the gamma WGT.**

| Genes | Annotation |
| --- | --- |
| RC2G0629600 | Integrase-type DNA-binding superfamily protein |
| RC4G0440541 | Integrase-type DNA-binding superfamily protein |
| RC2G0108831 | Integrase-type DNA-binding superfamily protein |
| RC6G0310091 | Integrase-type DNA-binding superfamily protein |
| RC3G0475921 | Leucine-rich repeat protein kinase family protein |
| RC2G0158821 | Mitogen-activated protein kinase kinase kinase 16 |
| RC5G0002581 | Mitogen-activated protein kinase kinase kinase 16 |
| RC3G0464821 | POX (plant homeobox) family protein |
| RC6G0244071 | Regulatory particle non-ATPase 10 |
| RC2G0099211 | Regulatory particle non-ATPase 10 |
| RC2G0686300 | Thioredoxin superfamily protein |
| RC4G0341500 | Thioredoxin superfamily protein |
| RC5G0048201 | Transmembrane kinase 1 |

**Supplementary Table 4. The annotation of 11 retained genes involved in both salt stress and water stress in *Rosa rugosa* after the gamma WGT.**

| Genes | Annotation |
| --- | --- |
| evm.model.Chr3.4960 | ARM repeat superfamily protein |
| evm.model.Chr3.3318 | ARM repeat superfamily protein |
| evm.model.Chr6.2199 | ARM repeat superfamily protein |
| evm.model.Chr2.292 | ARM repeat superfamily protein |
| evm.model.Chr7.1662 | MAP kinase 11 |
| evm.model.Chr3.3035 | MAP kinase 11 |
| evm.model.Chr2.997 | Mitogen-activated protein kinase 1 |
| evm.model.Chr6.1486 | Mitogen-activated protein kinase 1 |
| evm.model.Chr6.6697 | Protein kinase superfamily protein |
| evm.model.Chr5.833 | With no lysine (K) kinase 5 |
| evm.model.Chr4.1107 | With no lysine (K) kinase 5 |

**Supplementary Table 5. The annotation of 7 retained genes involved in both salt stress and water stress in *Rosa chinensis* after the gamma WGT.**

| Genes | Annotation |
| --- | --- |
| RC6G0579900 | ARM repeat superfamily protein |
| RC2G0108501 | ARM repeat superfamily protein |
| RC3G0476021 | E3 ubiquitin-protein ligase PUB24-like protein |
| RC5G0002581 | Mitogen-activated protein kinase 16 |
| RC5G0048571 | plant U-box 23 |
| RC2G0107441 | Protein kinase superfamily protein |
| RC6G0309321 | Protein kinase superfamily protein |

**Supplementary Table 6.** **Positively selected genes of *R. rugosa***.

| Gene pairs | Ka/Ks | Annotation |
| --- | --- | --- |
| evm.model.Chr2.1721__evm.model.Chr2.1827 | 99 | SPX domain-containing protein |
| evm.model.Chr2.1737__evm.model.Chr2.1846 | 99 | UDP-xylosyltransferase 2 |
| evm.model.Chr2.1805__evm.model.Chr2.1917 | 99 | Ubiquitin family protein |
| evm.model.Chr2.2166__evm.model.Chr2.2337 | 99 | Bax inhibitor-1 family protein |
| evm.model.Chr2.2256__evm.model.Chr2.2408 | 99 | DREB2A-interacting protein 2 |
| evm.model.Chr2.2262__evm.model.Chr2.2414 | 99 | winged-helix DNA-binding transcription factor family protein |
| evm.model.Chr2.2295__evm.model.Chr2.2447 | 99 | ARM repeat superfamily protein |
| evm.model.Chr2.2311__evm.model.Chr2.2590 | 99 | PEPTIDE TRANSPORTER 3 |
| evm.model.Chr2.2323__evm.model.Chr2.2541 | 99 | serine carboxypeptidase-like 50 |
| evm.model.Chr2.2764__evm.model.Chr2.2797 | 99 | peroxisomal 3-ketoacyl-CoA thiolase 3 |
| evm.model.Chr2.2766__evm.model.Chr2.2801 | 99 | hypothetical protein |
| evm.model.Chr2.2769__evm.model.Chr2.2804 | 99 | Galactose oxidase/kelch repeat superfamily protein |
| evm.model.Chr2.5277__evm.model.Chr2.5344 | 99 | HSP20-like chaperones superfamily protein |
| evm.model.Chr5.223__evm.model.Chr5.5 | 99 | HSP20-like chaperones superfamily protein |
| evm.model.Chr5.1492__evm.model.Chr5.1869 | 99 | TRM32-like protein |
| evm.model.Chr5.1506__evm.model.Chr5.1881 | 99 | Unknow function |
| evm.model.Chr5.1510__evm.model.Chr5.1886 | 99 | hypothetical protein |
| evm.model.Chr5.1543__evm.model.Chr5.1925 | 99 | 63 kDa inner membrane family protein |
| evm.model.Chr5.1548__evm.model.Chr5.1929 | 99 | carbonic anhydrase 1 |
| evm.model.Chr5.1556__evm.model.Chr5.1939 | 99 | dessication-induced 1VOC superfamily protein |
| evm.model.Chr5.1589__evm.model.Chr5.1970 | 99 | RNA polymerase Rpb7 N-terminal domain-containing protein |
| evm.model.Chr5.1590__evm.model.Chr5.1971 | 99 | histone H2A 12 |
| evm.model.Chr5.1650__evm.model.Chr5.2020 | 99 | UPSTREAM OF FLC protein |
| evm.model.Chr5.1663__evm.model.Chr5.2032 | 99 | RING/U-box superfamily protein |
| evm.model.Chr5.1664__evm.model.Chr5.2033 | 99 | ribosomal protein L23AB |
| evm.model.Chr5.1728__evm.model.Chr5.2118 | 99 | Glycosyl hydrolase family protein with chitinase insertion domain-containing protein |
| evm.model.Chr5.1755__evm.model.Chr5.2153 | 99 | DNA repair (Rad51) family protein |
| evm.model.Chr5.1778__evm.model.Chr5.2179 | 99 | Cysteine proteinases superfamily protein |
| evm.model.Chr5.1808__evm.model.Chr5.2206 | 99 | Transcription initiation factor TFIIE%2C beta subunit |
| evm.model.Chr2.1720__evm.model.Chr2.1826 | 99 | Adenine nucleotide alpha hydrolases-like superfamily protein |
| evm.model.Chr2.1722__evm.model.Chr2.1829 | 99 | NA polymerase II%2C Rpb4%2C core protein |
| evm.model.Chr2.1820__evm.model.Chr2.1934 | 99 | Bax inhibitor-1 family protein |
| evm.model.Chr2.2257__evm.model.Chr2.2409 | 99 | PRA1 (Prenylated rab acceptor) family protein |
| evm.model.Chr2.2270__evm.model.Chr2.2422 | 99 | INDH1(iron-sulfur protein required for NADH dehydrogenase) |
| evm.model.Chr2.2317__evm.model.Chr2.2585 | 99 | Class II aaRS and biotin synthetases superfamily protein |
| evm.model.Chr5.1487__evm.model.Chr5.1860 | 99 | outer plastid envelope protein 16-1 |
| evm.model.Chr5.1584__evm.model.Chr5.1965 | 99 | Concanavalin A-like lectin protein kinase family protein |
| evm.model.Chr5.1586__evm.model.Chr5.1967 | 99 | Myb/SANT-like DNA-binding domain protein |
| evm.model.Chr5.1708__evm.model.Chr5.2095 | 99 | Spo11/DNA topoisomerase VI%2C subunit A protein |
| evm.model.Chr5.1796__evm.model.Chr5.2194 | 99 | branched-chain alpha-keto acid decarboxylase E1 beta subunit |
| evm.model.Chr5.6640__evm.model.Chr5.7033 | 99 | DNA-binding storekeeper protein-related transcriptional regulator |
| evm.model.Chr5.6645__evm.model.Chr5.7028 | 99 | C-terminal cysteine residue is changed to a serine 1 |
| evm.model.Chr5.6655__evm.model.Chr5.7020 | 99 | cysteine-rich RLK (RECEPTOR-like protein kinase) 29 |
| evm.model.Chr5.1147__evm.model.Chr7.3855 | 99 | cell division control 6 |
| evm.model.Chr7.4939__evm.model.Chr7.4973 | 99 | hypothetical protein |
| evm.model.Chr2.2324__evm.model.Chr2.2540 | 99 | hypothetical protein |
| evm.model.Chr6.3503__evm.model.Chr6.3603 | 99 | G-type lectin S-receptor-like Serine/Threonine-kinase |
| evm.model.Chr1.1541__evm.model.Chr1.1571 | 34.982 | NAD(P)-linked oxidoreductase superfamily protein |
| evm.model.Chr5.1488__evm.model.Chr5.1859 | 14.0441 | Galactose oxidase/kelch repeat superfamily protein |
| evm.model.Chr1.2125__evm.model.Chr2.2049 | 10.3289 | CBS domain protein with a domain protein |
| evm.model.Chr4.3323__evm.model.Chr4.3870 | 7.1863 | Protein kinase superfamily protein |
| evm.model.Chr1.4308__evm.model.Chr7.1350 | 6.9827 | pantothenate kinase 2 |
| evm.model.Chr5.1784__evm.model.Chr5.2184 | 4.539 | Dof-type zinc finger DNA-binding family protein |
| evm.model.Chr2.2264__evm.model.Chr2.2416 | 2.7814 | Pentatricopeptide repeat (PPR) superfamily protein |
| evm.model.Chr5.1713__evm.model.Chr5.2101 | 2.762 | Nucleotide-sugar transporter family protein |
| evm.model.Chr2.3174__evm.model.Chr7.3181 | 2.2102 | glutamate receptor 2 |
| evm.model.Chr2.1736__evm.model.Chr2.1843 | 2.0018 | alpha-soluble NSF attachment protein 2 |
| evm.model.Chr5.102__evm.model.Chr5.56 | 2 | Protein kinase domain |
| evm.model.Chr1.4445__evm.model.Chr7.327 | 2 | DnaJ domain |
| evm.model.Chr6.3381__evm.model.Chr6.3453 | 1.9977 | / |
| evm.model.Chr6.3392__evm.model.Chr6.3465 | 1.996 | / |
| evm.model.Chr3.924__evm.model.Chr7.3864 | 1.8197 | Male sterility protein |
| evm.model.Chr5.1699__evm.model.Chr5.2081 | 1.7665 | Retrotransposon gag protein |
| evm.model.Chr2.1784__evm.model.Chr2.1895 | 1.5808 | Thioredoxin -like |
| evm.model.Chr5.1730__evm.model.Chr5.2124 | 1.4518 | / |
| evm.model.Chr5.1544__evm.model.Chr5.1926 | 1.366 | / |
| evm.model.Chr2.2296__evm.model.Chr2.2448 | 1.2848 | Poly(ADP-ribose) polymerase, regulatory domain |
| evm.model.Chr2.2265__evm.model.Chr2.2417 | 1.181 | Transcription initiation factor IIF, alpha subunit (TFIIF-alpha) |
| evm.model.Chr2.1802__evm.model.Chr2.1914 | 1.1797 | OTU-like cysteine protease |
| evm.model.Chr2.2793__evm.model.Chr2.2812 | 1.1679 | Lipoxygenase |
| evm.model.Chr2.2271__evm.model.Chr2.2423 | 1.1385 | Zein-binding |
| evm.model.Chr5.1772__evm.model.Chr5.2171 | 1.0974 | / |
| evm.model.Chr5.1496__evm.model.Chr5.1875 | 1.0809 | MED6 mediator sub complex component |
| evm.model.Chr6.3498__evm.model.Chr6.3616 | 1.0573 | Protein of unknown function (DUF1685) |
| evm.model.Chr2.2753__evm.model.Chr2.2993 | 1.0474 | Transposase, Mutator family |
| evm.model.Chr5.1677__evm.model.Chr5.2050 | 1.0416 | PPR repeat |
| evm.model.Chr2.2205__evm.model.Chr2.2356 | 1.0282 | Cupin superfamily protein |

**Supplementary Table 7. Gene number variation on the salt stress pathway across the three species, *Arabidopsis thaliana*, *R. rugosa*, *R. chinensis*.**

| Gene | *Arabidopsis thaliana* | *Rosa rugosa* | *Rosa chinensis* |
| --- | --- | --- | --- |
| 14-3-3 | 3 | 1 | 2 |
| ABI2 | 3 | 3 | 3 |
| GI | 1 | 1 | 1 |
| J3 | 2 | 2 | 2 |
| MAPK6 | 1 | 1 | 1 |
| PA | 2 | 2 | 3 |
| PKS24 | 1 | 1 | 1 |
| PKS5 | 1 | 1 | 1 |
| SOS2 | 2 | 1 | 1 |
| SOS3 | 5 | 1 | 1 |
| ScaBP8 | 6 | 1 | 1 |
| ScaBP1/2/3 | 5 | 1 | 1 |


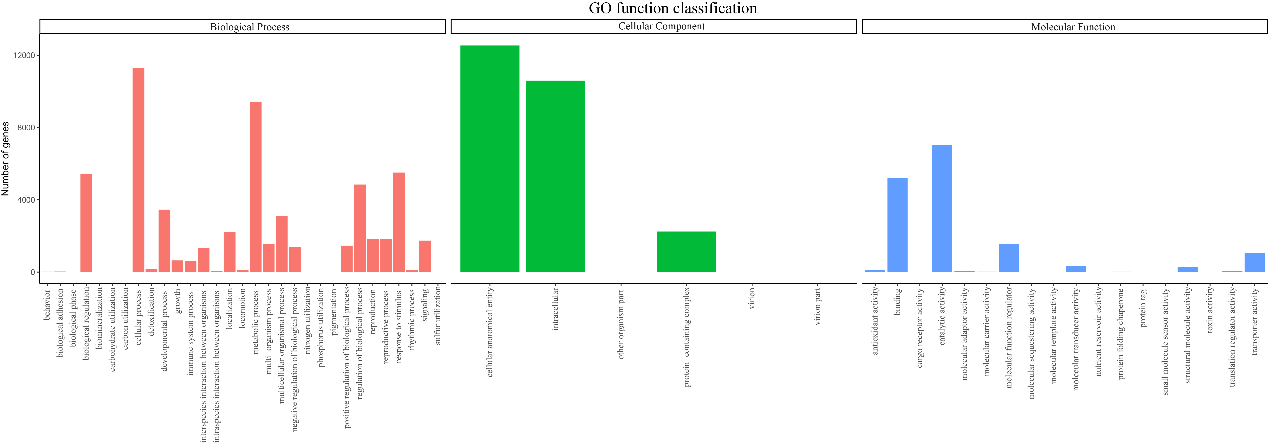


**Supplementary Fig.1 GO function classification of *R. rugosa* proteins predicted from orthovenn2 (https://orthovenn2.bioinfotoolkits.net/home).**


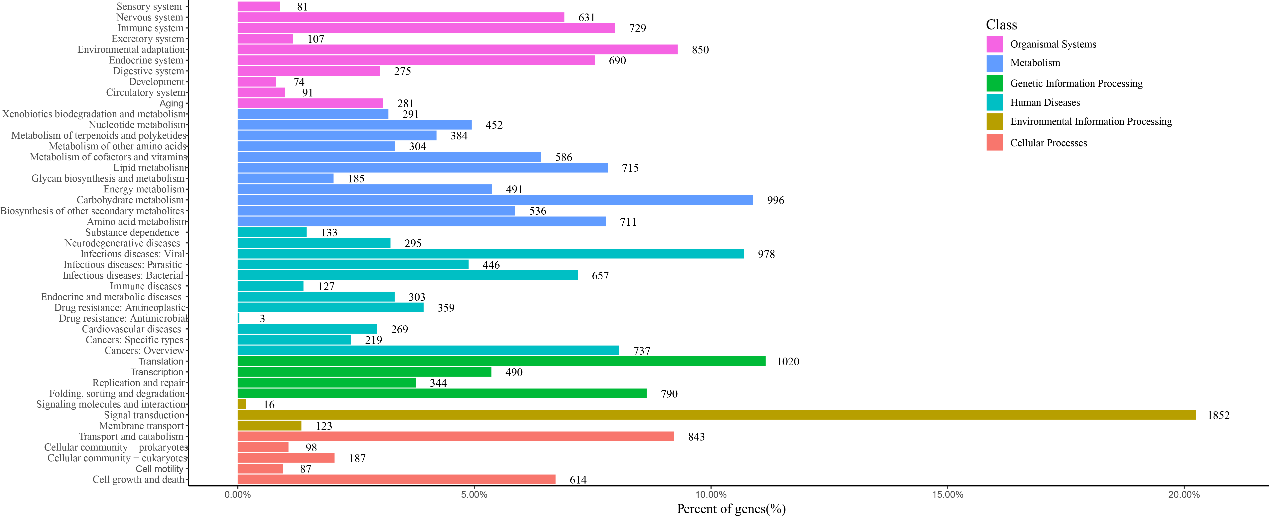


**Supplementary Fig. 2. Pathway analysis of *R. rugosa* proteins predicted from orthovenn2 (https://orthovenn2.bioinfotoolkits.net/home).**


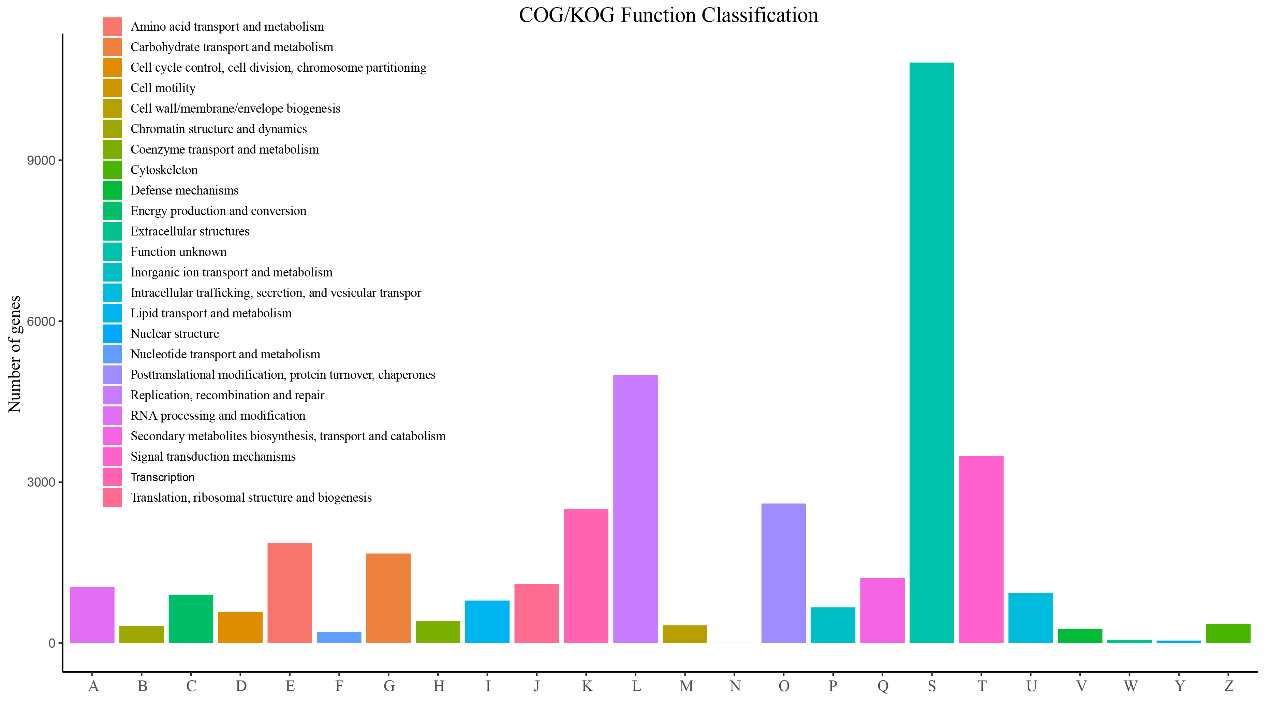


**Supplementary Fig.3. COG analysis of *R. rugosa* proteins predicted from orthovenn2 (https://orthovenn2.bioinfotoolkits.net/home).**


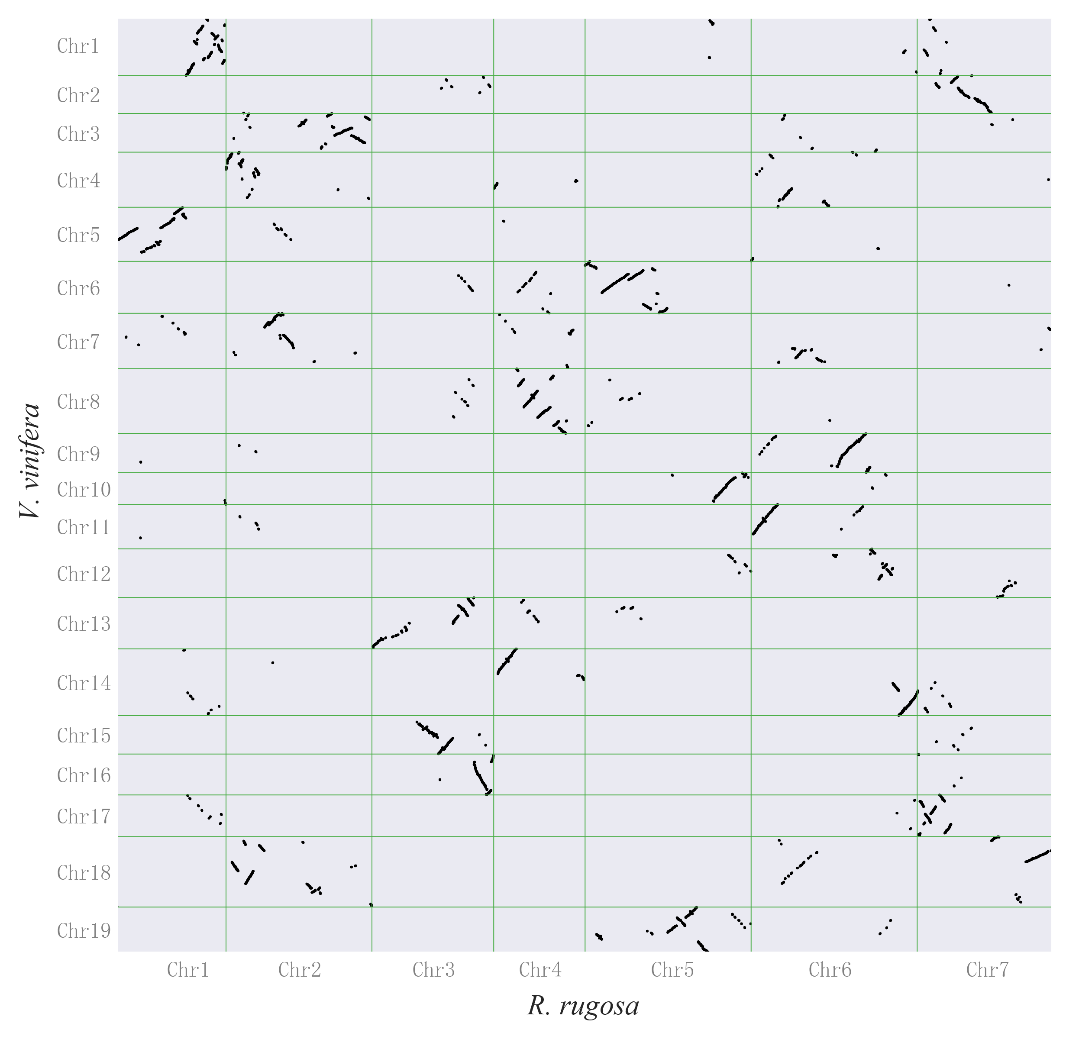


**Supplementary Fig.4. Whole-genomic dotplot showing the syntenic relationships between *R. rugosa* and grape (*V. vinifera*).**


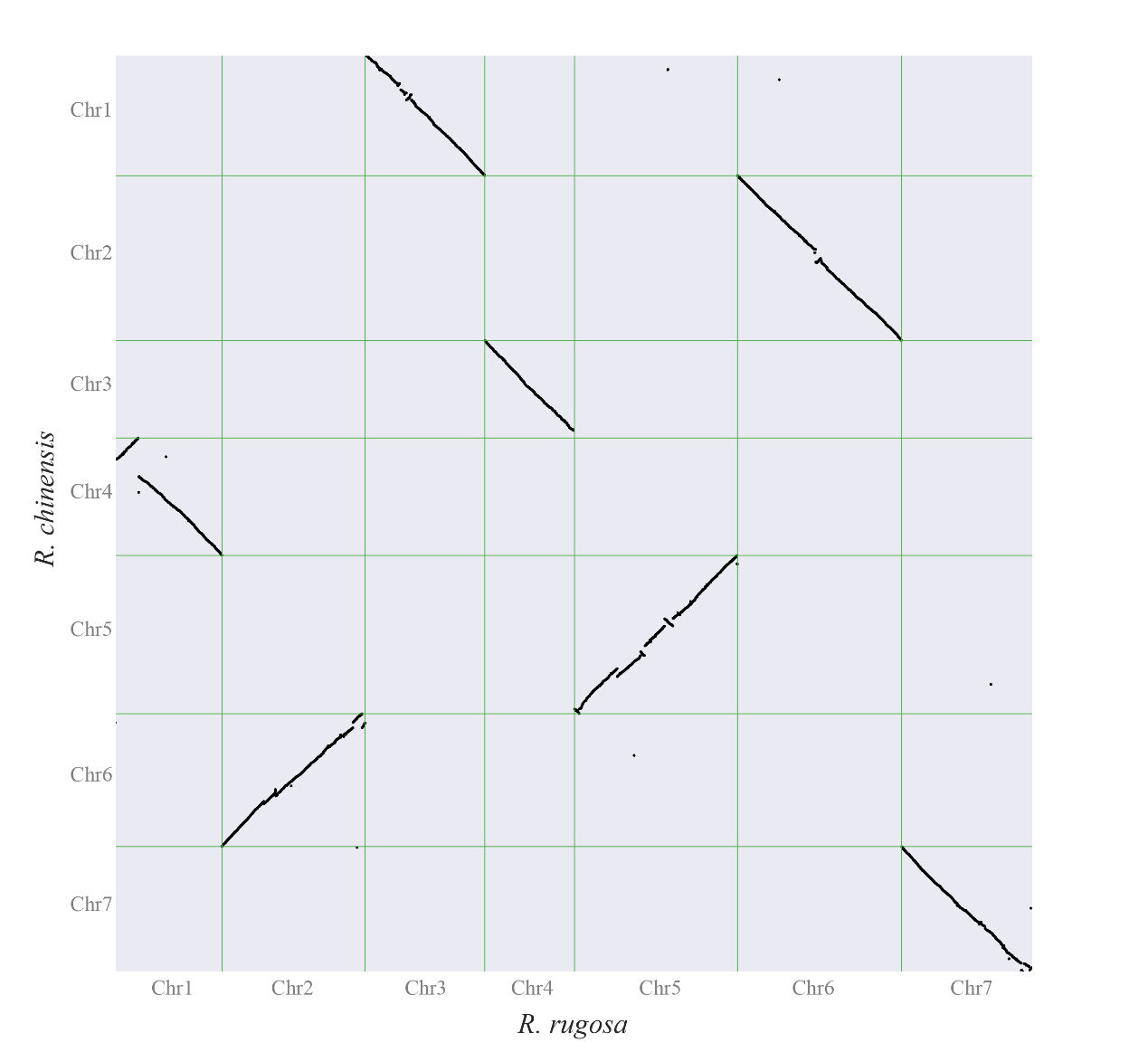


**Supplementary Fig.5. Whole-genomic dotplot showing the syntenic relationships between *R. rugosa* and**

***R. chinensis*.**


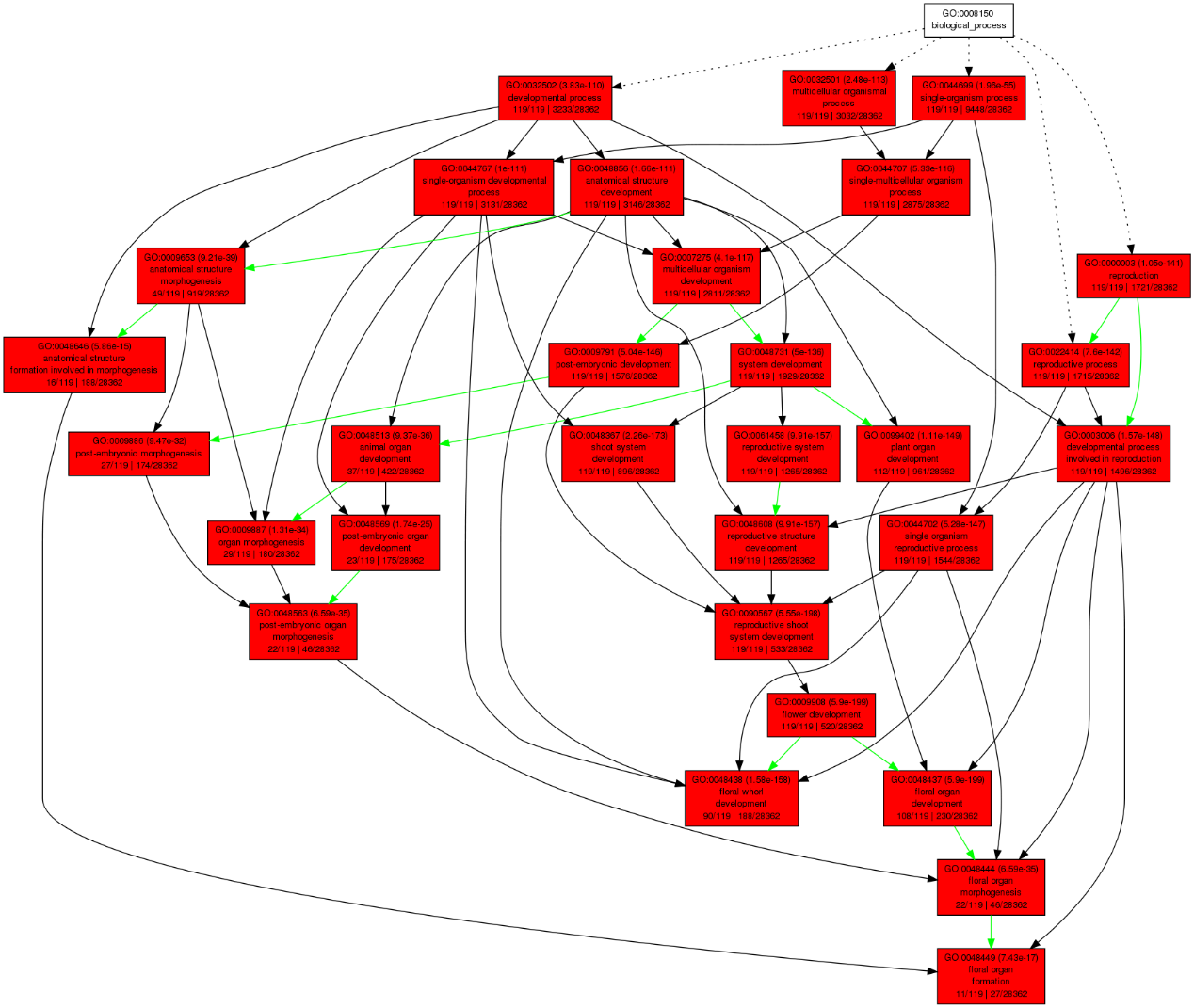


**Supplementary Fig. 6. The 119 retained genes after WGT were involved in floral development using *Arabidopsis* orthologs of *R. rugosa* genes.**


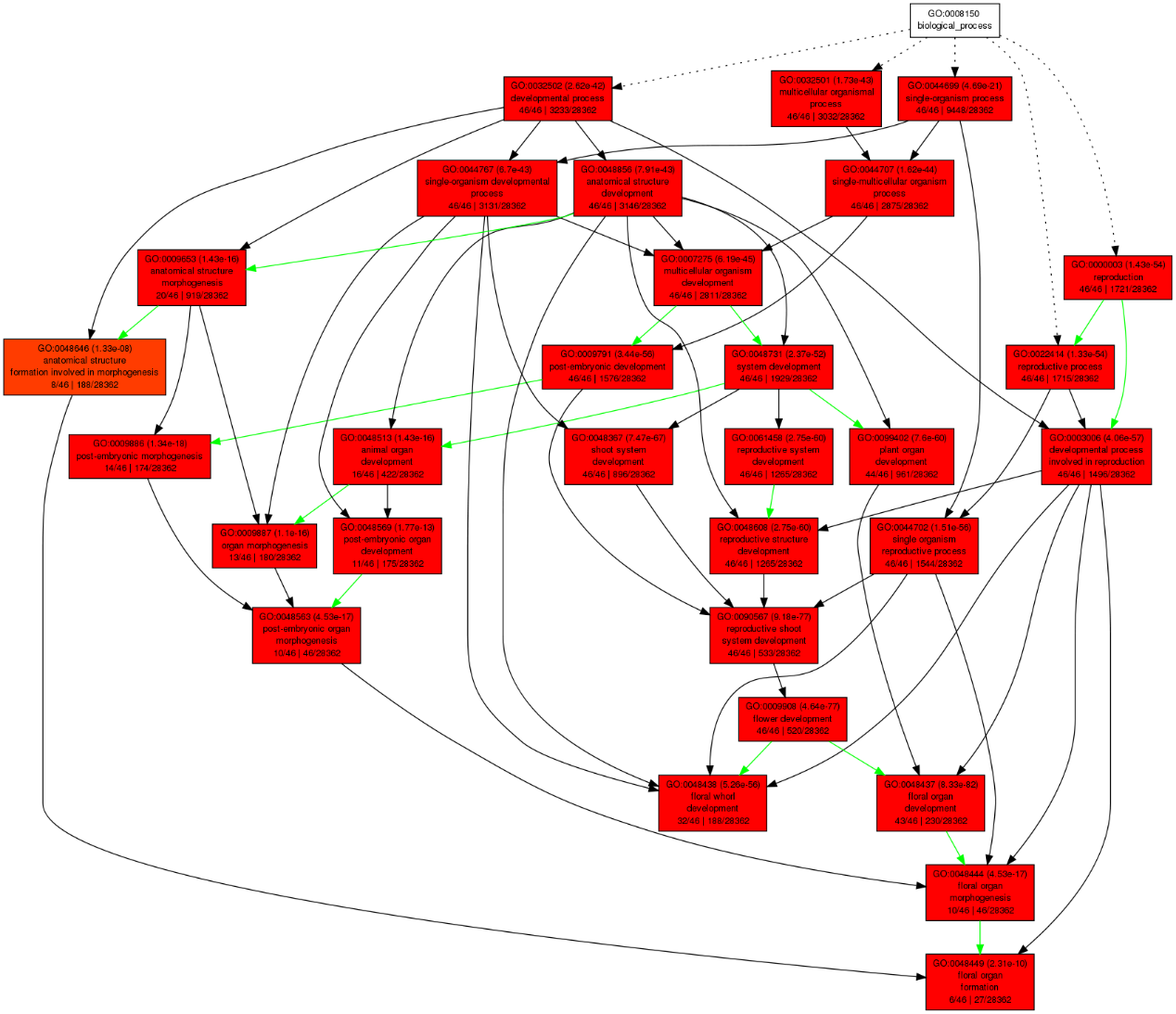


**Supplementary Fig. 7. The 46 retained genes after WGT were involved in floral development using *Arabidopsis* orthologs of *R. chinensis* genes.**


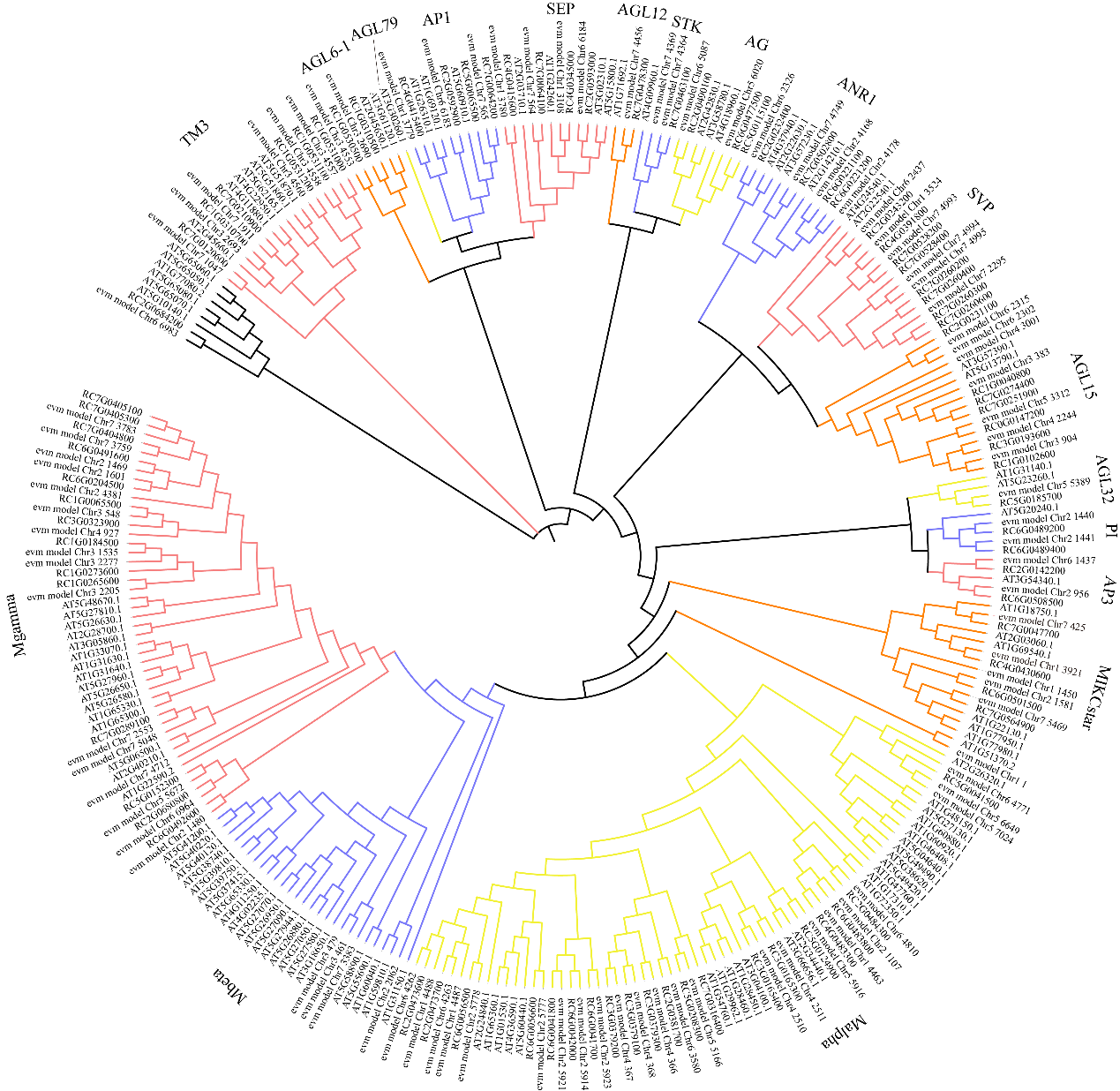


**Supplementary Fig. 8. The phylogenetic tree of MADS-box transcription factor genes from *R. rugosa*, *R. chinensis*, and *A. thaliana*.** Each subfamily of *MADS-box* was shown as branches with differently colors.

**Supplementary Fig. 9.** The *S*-locus in *R. rugosa* and other related Rosaceae plants. A, Schematic diagram of the *S*-locus in *Prunus* spp[1]. B, Schematic diagram of the *S*-locus in *Malus* and *Pyrus* spp[2]. C, The *S*-locus in *R. rugosa* composed of 667 Kb. D, Phylogeny of *S-RNase* from representative species. Orange: *R. chinensis[3].* Blue, strawberry (unpublished by Jianke Du, et al.).


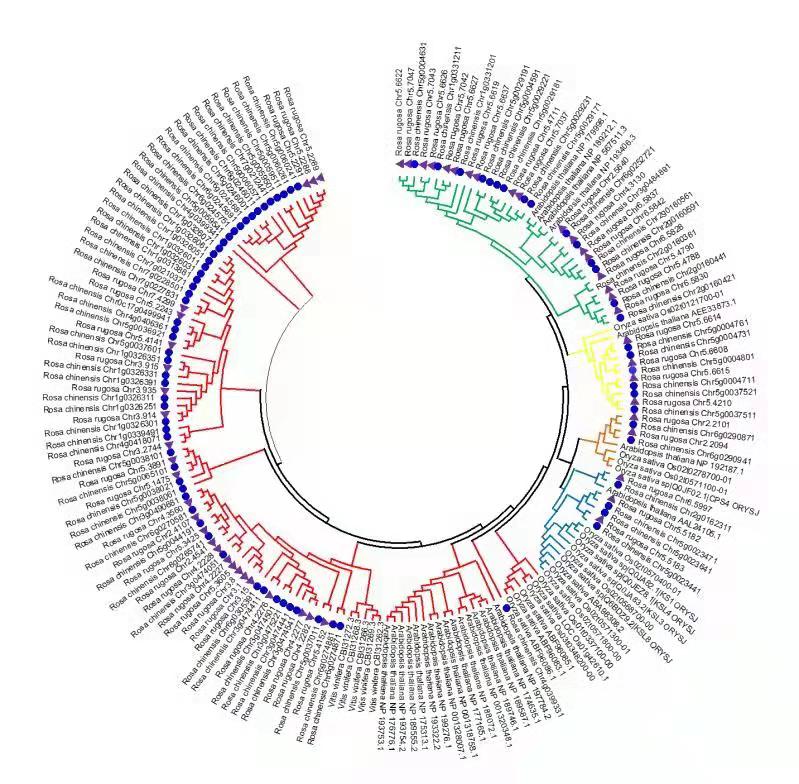


**Supplementary Fig. 10. The phylogenetic tree of terpene synthase genes from *R. rugose* and *R. chinensis*, as well as a few other plants.** Red line indicate the TPS-a subfamily, green for TPS-g subfamily, yellow for TPS-b subfamily, brown for TPS-c, blue for TPS-e/f.


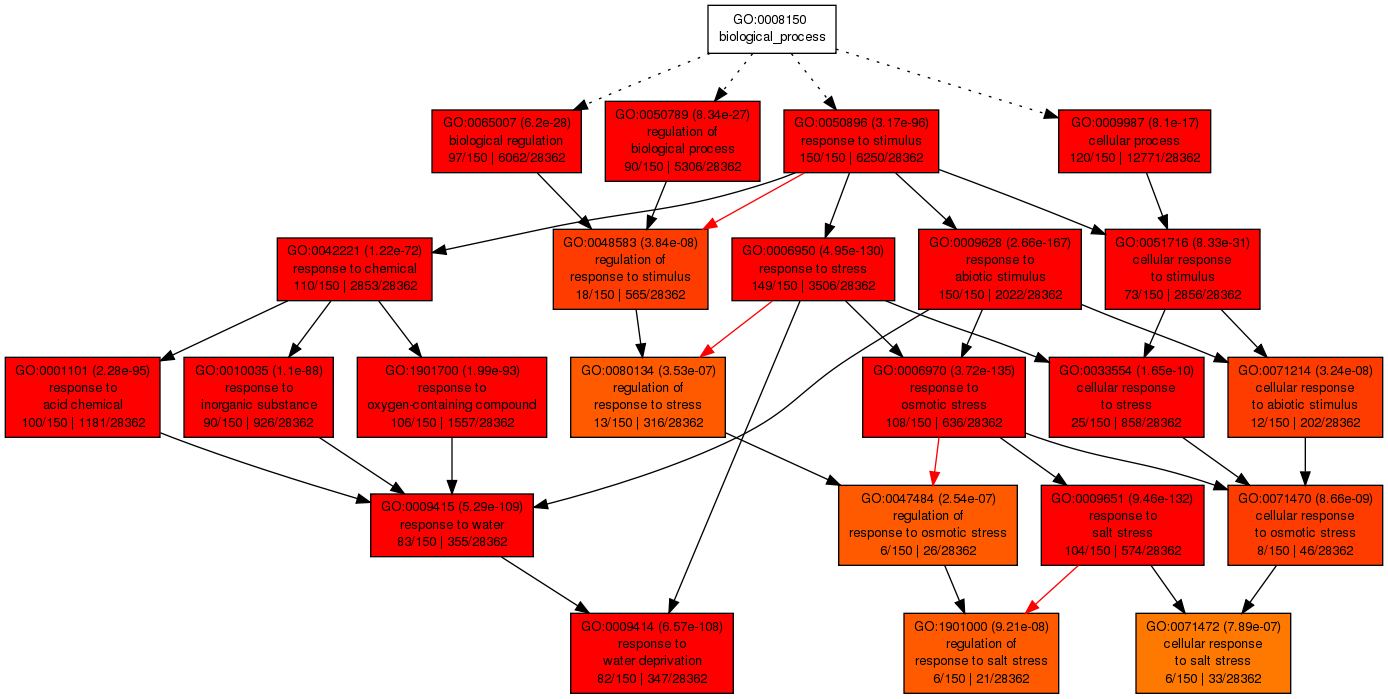


**Supplementary Fig. 11.** The agriGO modules of salt stress and water stress related genes, predicted using *Arabidopsis* orthologs of *R. chinensis* duplicated genes after the WGT.

**References**

1. Tsukamoto T, Hauck NR, Tao R, Jiang N, Iezzoni AF: **Molecular and genetic analyses of four nonfunctional S haplotype variants derived from a common ancestral S haplotype identified in sour cherry (Prunus cerasus L.)**. *Genetics* 2010, **184**(2):411-427.

2. Li K, Wang Y, Qu H: **RNA-Seq analysis of compatible and incompatible styles of Pyrus species at the beginning of pollination**. *Plant Mol Biol* 2020, **102**(3):287-306.

3. Vieira J, Pimenta J, Gomes A, Laia J, Rocha S, Heitzler P, Vieira CP: **The identification of the Rosa S-locus and implications on the evolution of the Rosaceae gametophytic self-incompatibility systems**. *Sci Rep* 2021, **11**(1):3710.
